# Who seeks treatment for gaming? Characteristics of young and adult patients seeking treatment for gaming disorder

**DOI:** 10.3389/fpsyt.2025.1629932

**Published:** 2025-08-12

**Authors:** Per Bore, Mitchell Andersson, Sara Nilsson, Kajsa Oehm, Matti Cervin, Anders Håkansson, Emma Claesdotter-Knutsson

**Affiliations:** ^1^ Section for Psychiatry, Department of Clinical Sciences Lund, Faculty of Medicine, Lund University, Lund, Sweden; ^2^ Clinical Addiction Research Unit, Malmö Addiction Center, Malmö, Sweden; ^3^ Child and Adolescent Psychiatry, Regional Outpatient Care, Lund, Sweden

**Keywords:** gaming disorder, internet gaming disorder, adolescents, youth, treatment seeking, clinical characteristics

## Abstract

**Background:**

Gaming disorder has recently been recognized as a psychiatric condition, yet the clinical characteristics of treatment-seeking individuals remain understudied. This study examined youth and adults seeking treatment at a specialized outpatient clinic in southern Sweden.

**Methods:**

A total of 107 individuals aged 12–49 years (M = 22.1, SD = 7.2) underwent comprehensive clinical interviews, psychosocial assessments, MINI diagnostic interview, and standardized self-report measures.

**Results:**

Most participants were male (94%), and 80% met diagnostic criteria for gaming disorder. The average age of symptom onset was 16.0 years (SD = 4.6), with a mean duration of 5.5 years (SD = 4.6). Weekly gaming time averaged 50 hours (SD = 12.0, range 0–126). Although participants reported low levels of gaming disorder symptoms (measured by GDT) and psychological distress (measured by CORE-OM and RCADS), but 69% showed significant functional impairments based on clinician ratings using GAF and CGAS. ADHD symptoms were uniquely positively associated with both gaming disorder severity (β=0.39, p < 0.001) and psychological distress (β=0.34, p < 0.001). Psychological distress also increased with age (β=0.38, p=0.002).

**Discussion:**

Although many received a clinical diagnosis, the sample reported low levels of gaming disorder symptoms. They reported relatively low psychological distress but demonstrated substantial functional impairment. This may reflect gaming’s role as both an avoidance strategy and a way to meet psychological needs.

**Conclusion:**

These findings suggest that impaired everyday functioning is a defining clinical feature of this group. Treatment should not only address gaming behavior but also support patients in improving functioning across important areas of life, such as school, work, and relationships.

## 1 Introduction

Gaming Disorder (GD) was added to International Classification of Diseases (ICD-11) as a psychiatric diagnosis in 2019 (1). The disorder is defined by a pattern of gaming behavior characterized by impaired control, prioritization of gaming over other activities, and persistence or escalation of gaming despite negative consequences. Further, the gaming must cause significant impairment in important areas of functioning. In 2013, internet gaming disorder (IGD) was included in the section “Conditions for Further Study” in the Diagnostic and Statistical Manual of Mental Disorders (DSM-5), and remains in this section in the DSM-5-TR (2, 3). This means that IGD is not currently recognized as a formal diagnosis within the DSM classification system. The ICD and DSM systems differ in their conceptualization of problematic gaming, highlighting a broader lack of consensus regarding its definition and clinical relevance. Researchers have criticized the development of both gaming disorder and IGD as premature due to limited evidence, its parallels with gambling disorder and the risk of pathologizing normal youth behavior (4). Critics emphasize the need for more robust research, such as studies with treatment-seeking individuals, rather than self-reported surveys in predominantly healthy populations (5, 6). Research suggests that self-reported symptoms of gaming disorder may be both over- and underreported (7), and participants in gaming surveys may intentionally provide inaccurate responses (8).

Studies based on non-clinical samples and online surveys have provided important insights. The estimated global point prevalence of gaming disorder is 1.96% and is most common in male adolescents and young adults (9). Gaming motives vary by gaming disorder severity, with motivations related to emotional escape showing a strong link to more severe difficulties (10). Various difficulties are associated with gaming disorder symptoms, such as suicidal ideation, sleep disturbances, poor academic performance, poor emotion regulation, loneliness, lower social ability and lower executive functioning (11–14). A review by Dullur, Krishnan (15) found an association between attention deficit hyperactivity disorder (ADHD) and gaming disorder, while Eltahir, Delfabbro (16) reported an elevated risk among individuals with autism spectrum disorder (ASD).

Few studies have investigated the clinical characteristics of patients seeking treatment for gaming disorder. Hyun, Han (17) reported among 263 patients, that 97% were male with a mean age of 20.4 years. Symptoms of depression and ADHD were positively correlated with gaming disorder severity. Torres-Rodríguez, Griffiths (18) included 31 male adolescents with internet gaming disorder, all had a comorbid psychiatric disorder. Most participants showed elevated distress levels on the Symptom Checklist-90, though adolescent-specific assessments indicated less severe problems. Granero, Fernandez-Aranda (19) examined 107 patients with gaming disorder, aged 14 to 60 years, of whom 92% were male. Those with greater gaming-related impairment were older, had a later onset of problematic gaming, higher levels of psychological distress and more dysfunctional personality profiles. A Swedish study of 69 patients with gaming disorder observed high rates of self-reported ADHD (56.1%), ASD (39.7%), and problematic gambling (27.9%) among young and adult participants (20).

Despite growing research on gaming disorder, there is a need for more studies focusing on treatment-seeking individuals and their characteristics. Existing studies often rely on small or narrowly defined samples and limited assessment methods. This constrains our understanding of how gaming disorder presents in real-world clinical populations. Our study addresses this gap by including all individuals seeking help at a specialized outpatient clinic, using minimal inclusion criteria to create a clinically representative sample. Through comprehensive assessment of gaming behavior, related problems, psychological distress, psychiatric comorbidities, and everyday functioning, we explore the clinical and psychosocial characteristics of this population of treatment-seeking youth and adults. We also aim to examine how these factors are associated to better understand gaming-related impairment. Given the exploratory nature of this study, no *a priori* hypotheses were specified. A protocol for the larger research project has been published (21).

## 2 Materials and methods

### 2.1 Procedure and participants

The study was conducted in routine care, at a psychiatric outpatient clinic specialized in gaming disorder in southern Sweden. The clinic opened in March 2022 and is open to all individuals over the age of 13. Patients access the clinic through self-referrals or referrals from healthcare providers, schools, or social services. The healthcare system in Sweden is mostly government-funded, and all visits to this clinic are free of charge.

Over a 30-month period, from March 2022 to August 2024, all patients who started an assessment were invited to participate. During this time, 138 patients had at least one visit to the clinic, but not all started an assessment. In total, 113 consented to participate, and the final sample consisted of 107 participants, see the Appendix for the CONSORT diagram. The study aimed to capture the full range of treatment-seeking patients; hence the inclusion and exclusion criteria were minimal. Inclusion criteria were: 1) age ≥ 13 and 2) seeking treatment for problematic gaming. Problematic gaming was broadly defined as gaming that, according to the participant or their relatives, negatively affected daily functioning, caused distress, or led to interpersonal conflict. This determination was made based on referral information and initial clinical screening prior to the structured diagnostic interviews. Exclusion criteria were: 1) somatic or psychiatric conditions that would contraindicate assessment, such as the need for inpatient care or severe intellectual disability, and 2) inability to read and communicate proficiently in Swedish. One individual was 12 years old at the time of the assessment but was invited into the study upon turning 13.

The assessment was comprehensive and included structured interviews and questionnaires over approximately three sessions (50 minutes each). Interviewers included licensed psychologists, a social worker, and a psychotherapist. The assessment was adjusted to the participant’s age, with some differences in measures for participants aged 13–15 and those aged 16 years+. The decision to use different measures for the 13–15 age group was to ensure measures that were appropriate for their age. Data were analyzed both for the total sample and stratified by age: 13–15, 16–19, and 20+. This stratification accounts for the use of different measures and that individuals in Sweden typically complete upper secondary education by age 19.

### 2.2 Measures

A structured interview captured detailed sociodemographic information, gaming behaviors, relationships, money spent on gaming, physical health, sleep, and eating habits. The games were categorized into five novel game genres (22): 1) Competitive games, which emphasize shorter competitive matches; 2) Story-driven games, offline experiences with a beginning and end; 3) Single Player Simulation Strategic Progression games (SimStrat), games focused on world-building or strategic gameplay; 4) Massively multiplayer online game Extended (MMOE), games with a possible social component and long-term play; and 5) Casual games, characterized by simple challenges with increasing difficulty, often without story. The Mini International Neuropsychiatric Interview (MINI) was used to assess mental disorders based on DSM-5 criteria (23). For participants aged 13-15, the equivalent MINI-KID was used (24). ADHD and ASD diagnoses were reported by participants during the clinical interview and verified by the clinician through review of the patient’s medical records. Clinicians rated everyday functioning using Global Assessment of Functioning (GAF) (25) and the Children’s Global Assessment Scale (CGAS) for participants under age 16 (26). Gaming disorder was diagnosed by the clinician conducting the assessment in consultation with the team, following the criteria in ICD-11. Participants who did not meet the full diagnostic criteria but showed a pattern of problematic gaming that posed an elevated risk of harm were classified as having “hazardous gaming.” This categorization followed ICD-11 guidelines, which define hazardous gaming as gaming behavior that increases the likelihood of negative physical or mental health outcomes, even if it does not meet the threshold for a formal gaming disorder diagnosis. Finally, participants completed a battery of self-report measures; see **Table 1** for a detailed description.

**Table 1:** Self-report measures.

Instrument	Purpose	Respondent	Items & scale	Score range & cut-off levels	Reference
Gaming Disorder Test (GDT)	Gaming disorder symptoms, ICD-11 criteria	All	4 items; 5-point Likert (1–5)	Total: 4–20 Cut-off: all items ≥ 4 or 5	(49)
Internet Gaming Disorder Scale – Short Form (IGDS9-SF)	Internet gaming disorder symptoms, DSM-5 criteria	All	9 items; 5-point Likert scale (1–5)	Total: 9–45 Cut-off: ≥32	(50, 51)
Gaming Addiction Identification Test (GAIT)	Problematic gaming behavior	13–15 years old, parent-report	15 items; 5-point Likert scale (0–4)	Total: 0–60 Cut-off: ≥19	(52)
Clinical Outcomes in Routine Evaluation – Outcome Measure (CORE-OM)	Psychological distress	16 years +	34 items; 5-point Likert scale (1–5)	Total: 0-136 Cut-off: M ≥1.5	(27, 53)
Revised Child Anxiety and Depression Scale (RCADS)	Psychological distress	13 – 15-year-old, self- and parent-report	47 items; 5-point Likert scale (0-4)	Total: 0-141 Cut-off: T-score ≥ 65	(54)
Ritvo and Asperger Diagnostic Scale (RAADS-14)	ASD symptoms	All	14 items; 4-point Likert scale (0-3)	Total: 0-56 Cut-off: ≥ 14	(55)
Autism Quotient (AQ)	ASD symptoms	13 – 15-year-old, parent-report	10 items; 4-point Likert scale (1-4)	Total: 0-40 Cut-off: ≥ 6	(56)
Adult ADHD Self-Report Scale Part A (ASRS)	ADHD symptoms	All	6 items; 5-point Likert scale (0-4)	Total: 0-24 Cut-off: ≥ 15	(57, 58)
Swanson, Nolan, and Pelham Scale (SNAP-IV)	ADHD symptoms	13 – 15-year-old, parent-report	30 items; 4-point Likert scale (0-3)	Total: 0-120 Cut-off: M ≥1.67, question 1–9 and 21-28	(59, 60).
Bergen Social Media Addiction Scale (BSMAS)	Problematic social media use	All	6 items; 5-point Likert scale (1-5)	Total: 6–30 Cut-off: ≥ 26	(61)
Alcohol Use Disorders Identification Test – Consumption (AUDIT-C)	Alcohol dependence	All	3 items; 5-point Likert scale (0-4)	Total: 0-12 Cut-off: ≥ 6; alcohol dependence	(62–64)
Drug Identification List	Substance use	All	List of 10 common substances. 5-point Likert scale (0–4)	Frequency of use	–
NODS-PERC	Problematic gambling	All	4 items; binary response (yes/no)	Total: 0-4 Cut-off: ≥1	(65)
Motives for Online Gaming Questionnaire (MOGQ)	Gaming motives	All	27 items; 5-point Likert scale (1–5)	Mean score for each motive, 1-5	(66)

### 2.3 Statistical analyses

Data were screened for missing values and evaluated for conformity to the assumptions of normality. Although some missing data were present at the measure level, item-level missingness within individual self-report measures was low. To allow for the calculation of total scores and descriptive statistics, missing values at the item level were handled using median imputation within each questionnaire. This approach ensured minimal data loss in descriptive summaries. Normality was assessed using the Kolmogorov-Smirnov test and not all variables were normally distributed. We visually inspected the data and analyzed skewness with Fisher-Pearson standardized moment coefficient of skewness. All the variables that were not normally distributed exhibited mild skewness with small differences between mean and median values, suggesting that deviation from normality was minimal. Descriptive statistics were calculated for all variables. Continuous variables were summarized using means, standard deviations, and for some variables, ranges. Categorical variables were described using frequencies and percentages. In the presence of outliers, we provided median and quartiles. We also reported the proportion of participants reaching clinical cut-off levels on questionnaires. Analyses were conducted for the total sample and stratified across the three predefined age groups.

Bivariate associations among the study variables were examined using Pearson correlation coefficients. Given that psychological distress was assessed using two instruments across age groups, CORE-OM scores were converted to T-scores using the mean and standard deviation reported in Connell, Barkham (27). A combination of CORE-OM and RCADS (parent report) scores was pooled into a single variable of psychological distress for the whole sample. To further explore the relationships between variables, multiple linear regression models were conducted. For the regression analyses, missing data at the measure level across time points were handled using multiple imputation by chained equations (MICE) in R. Five imputed datasets were generated using predictive mean matching and results were pooled using Rubin’s rules. These models included relevant covariates to adjust for potential confounders. The coefficients are presented as standardized regression coefficients. The statistical significance level was determined *a priori* at an α-level of *p* = 0.05.

### 2.4 Ethics

The study was approved by the Swedish Ethical Review Authority (DNr: 2021-05923-01; 2022-03583–02 and 2023-03083-02). Written informed consent was obtained from all participants aged 15 years and older. For participants aged 13–14 years, consent was obtained by their parents and the children assented. The study was conducted in accordance with the principles outlined in the Declaration of Helsinki. This study is part of a larger study registered at Clinical trials (Clinical gov number: NCT06018922).

## 3 Results

### 3.1 Sociodemographic characteristics

Of the 107 participants, 22 were aged 13–15 years (21%), 24 were aged 16–19 years (22%) and 61 were 20 years or older (57%). Most patients were male (94%). The mean age was 22.07 years (SD = 7.23), with the youngest participant being 12 years old and the oldest being 49 years old. All participants who were 19 years and younger lived at home, and among older participants, 43% lived with their parents, 38% on their own and 12% with a partner. Sociodemographic characteristics stratified by age group are summarized in **Table 2**.

**Table 2:** Participants’ demographics.

Variable	13–15 years (*n* = 22)	16–19 years (*n* = 24)	≥20 years (*n* = 61)	Overall (*N* = 107)
Gender				
Man/boy	21 (95%)	23 (96%)	57 (93%)	101 (94%)
Woman/girl	1 (5%)	1 (4%)	4 (7%)	6 (6%)
Living situation				
With parents	22 (100%)	24 (100%)	26 (43%)	72 (67%)
Own residence	0	0	23 (38%)	23 (22%)
Own residence with partner	0	0	7 (12%)	7 (7%)
Own residence with non-family	0	0	3 (5%)	3 (3%)
Other	0	0	2 (3%)	2 (2%)
Completed education				
Primary	NA	22 (92%)	59 (98%)	81 (96%)
Secondary	NA	3 (13%)	40 (67%)	43 (51%)
Higher education	NA	NA	8 (13%)	8 (10%)
Missing			1	1
Civil status				
Single	NA	21 (88%)	46 (75%)	67 (79%)
In a relationship/married	NA	3 (13%)	15 (25%)	18 (21%)
Employment status				
Studying	NA	19 (79%)	18 (30%)	37 (44%)
Unemployed	NA	3 (13%)	27 (44%)	30 (35%)
Working	NA	0	12 (20%)	12 (14%)
Job placement	NA	1 (4%)	3 (5%)	4 (5%)
Other (e.g., sick leave)	NA	1 (%)	1 (2%)	2 (2%)
Route of referral				
Self- referral	1 (5%)	3 (13%)	36 (59%)	40 (37%)
Parent	18 (82%)	13 (54%)	10 (16%)	41 (38%)
Partner	0	0	4 (7%)	4 (4%)
Professionals*	3 (14%)	8 (33%)	11 (18%)	22 (21%)

*Professionals from school, healthcare or social services.

### 3.2 Gaming disorder

In the sample, 80% were diagnosed by a clinician with gaming disorder according to the ICD-11 criteria. The remaining participants did not meet the threshold for significant functional impairment but displayed problematic gaming behavior, these individuals were classified as having hazardous gaming, see **Table 3**. The mean age of onset for problematic gaming was 15 years, with an average of 5 years before seeking treatment. Many of the participants began playing between the ages of 5 to 6 (23%) or 10 to 12 (29%), the mean age of gaming debut was 10.0 years (SD = 4.8), see **Table 4**.

**Table 3:** Psychiatric co-morbidity among treatment-seekers.

Psychiatric diagnosis	Measure	Missing *n*	13–15 years *n* = 22	16–19 years *n* = 24	≥20 years *n* = 61	Overall *N* = 107
Depressive disorders	MINI	6	1 (6%)	2 (11%)	16 (28%)	19 (19%)
Previous depressive episode	MINI	6	5 (23%)	1 (5%)	27 (47%)	33 (31%)
ADHD	Interview	0	8 (36%)	7 (29%)	9 (15%)	24 (22%)
ASD	Interview	0	5 (23%)	1 (4%)	9 (15%)	15 (14%)
Anxiety disorders	MINI	7	2 (13%)	1 (5%)	14 (24%)	17 (17%)
Suicide ideation	MINI	6	0	0	2 (3%)	2 (2%)
Psychosis	MINI	7	0	0	2 (4%)	2 (2%)
PTSD	MINI	7	0	0	1 (2%)	1 (1%)
Anorexia/Bulimia	MINI	7	0	0	0	0
OCD	MINI	7	0	0	0	0
Any psychiatric diagnosis			11 (50%)	10 (42%)	35 (57%)	55 (51%)

Valid percentages are presented. ADHD, Attention deficit hyperactivity disorder; ASD, Autism spectrum disorder; PTSD, post-traumatic stress disorder; OCD, obsessive compulsive disorder.

**Table 4:** Gaming behavior.

Variable	13–15 years (*n* = 22)	16–19 years (*n* = 24)	≥20 years (*n* = 61)	Overall (N = 107)
Weekly gaming time, *M* (SD)	46.2 (25.0)	41.4 (16.7)	54.1 (24.6)	49.6 (23.5)
Missing	1	0	0	1
Most popular games, *n* (%)				
League of Legends	2 (9%)	5 (21%)	17 (28%)	24 (22%)
Counter-Strike	3 (14%)	8 (33%)	10 (16%)	21 (20%)
World of Warcraft	0	0	15 (25%)	15 (14%)
Minecraft	6 (27%)	4 (17%)	5 (8%)	15 (14%)
Fortnite	8 (36%)	2 (8%)	1 (2%)	11 (10%)
Valorant	3 (14%)	3 (13%)	3 (5%)	9 (8%)
Roblox	6 (27%)	2 (8%)	0	8 (8%)
Most popular genres, *n* (%)				
Competitive	15 (68%)	18 (75%)	38 (62%)	71 (66%)
Story driven	1 (5%)	1 (4%)	11 (18%)	13 (12%)
SimStrat	0	6 (25%)	10 (16%)	16 (15%)
MMOE Extended	11 (50%)	8 (33%)	29 (48%)	48 (45%)
Casual	7 (32%)	3 (13%)	5 (8%)	15 (14%)
Money spent on gaming (SEK), *Mdn* [Q1-Q3]	50 [0-130]	0 [0-335]	150 [0-463]	100 [0-300]
Games, *n* (%)	5 (25%)	7 (33%)	22 (39%)	34 (35%)
Skins, *n* (%)	7 (35%)	6 (29%)	19 (33%)	32 (33%)
Loot boxes, *n* (%)	0	3 (14%)	4 (7%)	7 (7%)
Subscriptions, *n* (%)	3 (15%)	1 (5%)	3 (5%)	7 (7%)
Pay-to-win, *n* (%)	0	0	2 (4%)	2 (2%)
Equipment, *n* (%)	0	0	1 (2%)	1 (1%)
In-game currency, *n* (%)	1 (5%)	0	0	1 (1%)
Missing	2	3	4	9
Spent any money on gaming, *n* (%)	13 (62%)	10 (42%)	39 (65%)	62 (59%)
Missing	1	0	1	2
Spent 1000 SEK or more on gaming, *n* (%)	0	3 (13%)	7 (12%)	10 (10%)
Missing	1	0	1	2
Age of gaming problem onset, *Mdn* [Q1-Q3]	12 [11-13]	15 [13-16]	17 [15-19]	15 [13-18]
Time since onset, *Mdn* [Q1-Q3]	2 [1-3]	3 [2-4]	7 [5-12]	5 [2-8]
Missing	9	12	19	40

Self-reported symptom severity of gaming disorder was overall low but varied between age groups. On GDT, the mean score was 13.2 (SD = 4.8) and 30% of the participants scored above the clinical cut-off. On IGDS9-SF, the mean score was 26.9 (SD = 8.5), and 35% scored above the clinical cut-off score of ≥ 32. When parents reported their children’s gaming disorder symptoms using GAIT, 95% were scored as above the clinical cut-off level of 19, and the mean was 40.3 points (SD=9.0). See **Table 5** for more information.

**Table 5:** Self-report questionnaires.

Variable	13–15 years (*n* = 22)	16–19 years (*n* = 24)	≥20 years (*n* = 61)	Overall (*n*= 107)
Diagnosed GD, n (%)	18 (86%)	16 (67%)	50 (83%)	84 (80%)
GDT, *M* (SD)	11.4 (4.9)	10.9 (4.1)	14.7 (4.5)	13.2 (4.8)
Above cut-off, *n* (%)	3 (15%)	3 (13%)	25 (43%)	31 (30%)
Missing	2	0	3	5
IGDS9-SF, *M* (SD)	22.1 (7.4)	22.7 (5.9)	30.3 (8.3)	26.9 (8.5)
Above cut-off ≥ 32, *n* (%)	2 (11%)	2 (8%)	31 (54%)	35 (35%)
Missing	3	0	4	7
GAIT, *M* (SD)	40.3 (9.0)	NA	NA	NA
Above cut-off, *n* (%)	20 (95%)			
Missing	2			
BSMAS, *M* (SD)	9.3 (3.8)	10.9 (3.9)	12.8 (5.0)	11.6 (4.7)
Above cut-off ≥ 26, *n* (%)	0	0	1 (3%)	1 (1%)
Missing	6	10	21	37
CORE-OM, *M* (SD)	NA	10.0 (3.5)	16.4 (6.2)	14.5 (6.3)
Moderate distress ≥ 15, *n* (%)	NA	1 (5%)	33 (61%)	34 (45%)
Missing	NA	2	7	9
RCADS, T-score (parent-rated)				
Depression & Anxiety, *M* (SD)	49.5 (10.7)	NA	NA	NA
Above cut-off ≥ 65, *n* (%)	1 (5%)	NA	NA	NA
Subscale anxiety, *M* (SD)	45.1 (9.7)	NA	NA	NA
Subscale depression, *M* (SD)	62.2 (13.1)	NA	NA	NA
Missing	4			
RCADS, T-score (child-rated)				
Depression & Anxiety, *M* (SD)	45.5 (11.3)	NA	NA	NA
Above cut-off ≥ 65, *n* (%)	2 (11%)	NA	NA	NA
Subscale anxiety, *M* (SD)	44.1 (10.7)	NA	NA	NA
Subscale depression, *M* (SD)	51.6 (12.2)	NA	NA	NA
Missing	5			
ASRS, *M* (SD)	10.0 (4.9)	10.9 (4.9)	13.2 (5.1)	12.2 (5.1)
Above cut-off ≥ 15, *n* (%)	3 (25%)	6 (25%)	24 (43%)	33 (36%)
Missing	10	0	5	15
SNAP-IV				
Inattention, *M* (SD)	16.2 (7.1)	NA	NA	NA
Hyperactivity/Impulsivity *M* (SD)	8.1 (7.2)	NA	NA	NA
Oppositional DD, *M* (SD)	11.0 (6.7)	NA	NA	NA
Above cut-off ADHD *M*≥ 1.67, *n* (%)	14 (74%)	NA	NA	NA
Missing	3			
RAADS-14, *M* (SD)	15.4 (16.8)	14.7 (9.6)	15.1 (10.1)	15.0 (10.8)
Above cut-off ≥ 14, *n* (%)	5 (46%)	14 (58%)	31 (55%)	50 (55%)
Missing	11	0	5	16
AQ, *M* (SD)	25.1 (4.1)	NA	NA	NA
Above cut-off ≥ 6, *n* (%)	8 (42%)	NA	NA	NA
Missing	3			
AUDIT-C, *M* (SD)	0.1 (0.3)	1.5 (2.0)	2.6 (2.0)	1.8(2.0)
Alcohol dependence ≥ 6, *n* (%)	0	1 (4%)	4 (7%)	5 (5%)
Missing	0	0	4	4
DUDIT				
Use now (ex. tobacco), *n* (%)	0	1 (4%)	4 (7%)	5 (5%)
Used before (ex. tobacco), *n* (%)	0	0	3 (5%)	3 (3%)
Use now tobacco, n (%)	0	0	17 (29%)	17 (16%)
Missing	0	0	2	2
NODS-PERC				
Risk-gambling ≥ 1, *n* (%)	0	11 (46%)	15 (27%)	26 (26%)
Missing	6	0	5	5
GAF, *M* (SD)	56.2 (11.4)	58.0 (9.5)	54.3 (12.5)	55.5 (11.7)
Score ≤ 60	13 (62%)	17 (71%)	42 (70%)	72 (69%)
Score ≤ 70	19 (91%)	21 (88%)	54 (90%	94 (90%)
Missing	0	0	1	1

Missing participant responses were imputed using the participant’s median score for the scale, provided at least one response was available. GAF, Global assessment of functioning; AUDIT-C, Alcohol use disorder identification test-consumption; BSMAS, Bergen Social Media Abuse Scale; DUDIT, Drug use disorder identification test; NODS-PERC, National Opinion Research Center DSM Screen for Gambling Problems - Preoccupation, Escape, Chasing and Risked Relationships measure; ASRS, Adult ADHD Self-Report Scale; SNAP-IV, Swanson, Nolan, and Pelham Rating Scale; RCADS, Revised Children’s Anxiety and Depression Scale; RAADS, Ritvo Autism Asperger Diagnostic Scale; CORE, Clinical Outcomes in Routine Evaluation; GAIT, Gaming Addiction Identification Test.

### 3.3 Gaming behavior

The participants reported playing video games for an average of 49.6 hours per week (SD = 23.5, range: 0 to 126) and played on computers (94%). The most common games were League of Legends (22%), Counter-Strike (20%), World of Warcraft (14%), Minecraft (14%), and Fortnite (10%). Thus, participants primarily played competitive games (e.g., Counter-Strike, League of Legends, Fortnite) and MMOE extended games (e.g., Minecraft, World of Warcraft). On the self-reported measure of gaming motives (MOGQ), participants across all age groups identified recreation as their primary motivation for gaming (M=4.30, SD=0.85), followed by escapism (M=2.98, SD=1.31) and coping (M=2.71, SD=0.86).

The median amount of money spent on gaming the last month was 100 SEK (~$10 USD). Most participants reported spending money on new games (35%), others spent money on in-game cosmetic items, such as skins (33%), and 7% reported purchasing loot boxes. In the sample, 10% spent more than 1000 SEK (~$100 USD) the last month. One participant had spent 6500 SEK (~$650 USD) during the last month on loot-boxes, which was substantially higher than the sample median. See **Table 4** for more information about gaming behavior.

### 3.4 Other screen behaviors

On average, participants reported spending 27.3 hours per week (SD = 18.5, range: 0–126 h) on non-gaming screen activities. On the self-report instrument BSMAS, evaluating problematic social media use, the mean score was 11.6 (SD = 4.7). Only one participant score above the cut off indicating a higher risk of problematic social media use.

### 3.5 Mental and neurodevelopmental disorders

Half of the participants (51%) met the criteria for at least one mental disorder, either through clinician-administered MINI or MINI-KID interview or by having a previously established diagnosis of ASD or ADHD, which they self-reported and which was verified in their medical records. The most common disorders were ADHD (22%), major depressive disorder (19%), and ASD (14%). Additionally, 31% reported experiencing previous depressive episodes. The frequency of the disorders is presented in **Table 3**.

Self-reported psychological distress varied across age groups. On CORE-OM, participants aged 20 and older reported greater psychological distress (M= 16.4, SD= 6.2) than 16–19-year-olds (M= 10.0, SD= 3.5), and a higher portion reached the cutoff for moderate-to-severe psychological distress (61% vs. 5%). For participants 13–15 years old, their self-reported psychological distress on RCADS was a T-score of 45.5 (SD=11.3), which is below the mean of peers. Parents reported their children’s psychological distress with a mean T-score of 49.5 (SD = 10.7), rising to 62.5 (SD = 13.1) on the depression subscale. Additionally, 11% of children and 5% of parents reported T-scores in the clinical range. In the entire sample, 36 participants (34%) scored above the clinical cut-off for psychological distress, see **Table 5**.

Self-reported symptoms of ASD were common across all age groups, with high variability in severity. The average RAADS-14 score was 15.0 (SD = 10.8) and 55% of participants exceeded the cut-off threshold. Among participants aged 13–15, 42% had parent-reported AQ scores above the cutoff. Self-reported ADHD symptoms were also high, 43% of the participants 20 years and older and 25% of participants 13–19 years old were above the cut-off on ASRS. Parents reported higher rates, with 74% of children meeting the ADHD cut-off on SNAP-IV.

Risk for alcohol dependence, screened with AUDIT-C, was detected in 5% of the sample. Regarding substance use, 5% reported current use, all involving cannabis and one participant also using other substances. Additionally, 37% of the sample had previously tried or used substances, with cannabis being the most common. For gambling, 26% of the sample endorsed at least one item on the NODS-PERC, suggesting potential gambling problems. During the structured-interview, 15 participants (14%) reported gambling with money or in-game items in the past month, while 31 (29%) indicated they had tried it at least once.

### 3.6 Everyday functioning and social life

Everyday functioning, as assessed by clinicians using GAF and CGAS, resulted in a mean score of 55.5 (SD = 11.7). In total, 69% had a score under 60, indicating significant problems with daily functioning. Among participants aged 20 years and older, 44% were unemployed, 5% were in work placement programs, 30% were studying, and 18% were employed. Of those 20 years and older, 67% reported having completed elementary school with passing grades. The participants aged 16–19 years were at the age when they should be attending secondary school; of these, 92% had passing grades from primary school. When the parents of participants aged 13–15 were asked about their children’s school performance, 36% reported that the child were meeting their academic goals and had passing grades. Most participants reported having multiple friends both online and offline. The distribution of time spent playing with friends online versus alone was bimodal, with one peak at lower proportions, indicating that many participants primarily play alone, and another at higher proportions, suggesting that others spend most of their time playing multiplayer games. Most participants reported being satisfied with their friendships both in-game and outside of gaming. However, only 48% said they spent at least one hour per week meeting friends outside of gaming. The lack of in-person social interaction was most pronounced among participants aged 13-15, with 29% engaging in in-person social interactions for at least one hour per week.

### 3.7 Physical health

Mean BMI was 23.6 (SD = 6.4), which is within the normal range but with a large variability across the sample. Participants reported an average of 7.5 hours of sleep per night (SD = 1.6), 40% had troubles falling asleep, and 17% frequently woke up during the night. Physical activity per week varied: 60% of participants reported less than the recommended 150 minutes of physical activity per week, while 37% met or exceeded the recommendations. Notably, 8% reported no physical activity at all.

### 3.8 Correlation and regression analyses

The interrelation of different variables was examined using both correlation and regression analyses. Gaming disorder symptom severity was strongly correlated with psychological distress (r = 0.51, *p* < 0.001), measured with CORE-OM and RCADS, and a moderate association was found with weekly gaming time (r = 0.28, p = 0.005). Everyday functioning also demonstrated a significant correlation with both psychological distress (r = 0.29, p = 0.004) and weekly gaming time (r = 0.29, p = 0.003). Correlations are presented in **Table 6**.

**Table 6:** Correlations between gaming disorder symptoms, distress, and everyday functioning, and other variables.

Key variable	Gaming disorder symptoms	Psychological distress	Everyday functioning
Gaming disorder symptoms	1		
Psychological distress	0.51 (p <0.001)*	1	
Everyday functioning	0.11 (p=0.257)	0.29 (p=0.004)*	1
Secondary variables			
Age	0.28 (p=0.004)*	0.57 (p <.001)*	0.20 (p=0.37)
Weekly game time	0.28 (p=0.005)*	0.18 (p=0.084)	0.29 (p=0.003)*
Problem gaming onset	0.48 (p <.001) *	0.52 (p <.001)*	0.11 (p=0.341)
Gaming debut	0.20 (p=0.054)	0.40 (p <.001)*	0.10 (p=0.350)
ASRS	0.55 (p <.001)*	0.55 (p <.001)*	0.25 (p=0.017)*
SNAP-IV	-0.25 (p=0.326)	0.38 (p=0.157)	0.30 (p=0.225)
RAADS-14	0.13 (p=0.213)	0.30 (p=0.006)*	0.29 (p=0.006)*
AQ	0.48 (p=0.049)*	0.53 (p=0.043)*	0.35 (p=0.158)
Audit-C	0.14 (p=0.168)	0.37 (p <.001)*	-0.05 (p=0.665)
Bergen social media scale	0.26 (p=0.029)*	0.24 (p=0.053)	-0.01 (p=0.948)
Nods-perc	-0.13 (p=0.223)	0.02 (p=0.820)	-0.03 (p=0.766)
Money spent in games	0.08 (p=0.401)	0.04 (p=0.683)	-0.17 (p=0.087)
% gaming with friends	-0.28 (p=0.005)*	-0.23 (p=0.027)*	-0.15 (p=0.138)
Time with friends, h/week	0.04 (p=0.728)	-0.01 (p=0.862)	-0.33 (p=0.001)*
Satisfied, friends outside games	-0.24 (p=0.017)*	-0.27 (p=0.011)*	-0.17 (p=0.088)
Sleep	0.07 (p=0.482)	-0.05 (p=0.607)	0.20 (p=0.041)*
Physical activity per week	-0.18 (p=0.070)	-0.13 (p=0.220)	-0.25 (p=0.011)*
BMI	0.03 (p=0.809)	0.42 (p <.001)*	0.16 (p=0.117)

ASRS, Adult ADHD Self-Report Scale; SNAP-IV, Swanson, Nolan, and Pelham Questionnaire – Version IV; RAADS-14, Ritvo Autism Asperger Diagnostic Scale – 14 items; AQ, Autism Spectrum Quotient; AUDIT-C, Alcohol Use Disorders Identification Test – Consumption; NODS-PERC, National Opinion Research Center DSM-IV Screen for Gambling Problems - Preoccupation, Escape, Risked relationships and Chasing losses.The * indicates that the p-value is statistically significant. The bold values are the values that are significant.

To identify unique associations, multiple linear regressions were conducted after performing multiple imputation for missing data. In the first model gaming disorder symptom severity was used as the outcome variable and age, psychological distress, everyday functioning, time spent gaming, onset of problematic gaming, ASRS and RAADS-14 as explanatory variables. In the first model, ADHD symptoms emerged as the strongest contributor (β=0.394, *p*<0.001) with the model explaining 39% of the variance. A similar model was created with psychological distress as the dependent variable. The significant explanatory variables were age (β=0.383, *p*=0.002), ADHD symptoms (β=0.336, < 0.001), onset of problematic gaming (β=0.214, *p*=0.019) and ASD symptoms (β=0.176, p=0.023), with the model explaining 61% of the total variance. Finally, a model with everyday functioning as the outcome variable showed that only weekly game time (β = 0.249, p =0.013) was a significant explanatory variable, and the model only accounted for 15% of the variance. Pooled results from the regression models are presented in **Table 7**.

**Table 7:** Pooled results from multiple linear regression models.

	Variables	Standardized beta [β]	95% CI	P
Model 1 Gaming disorder symptoms	Age	-0.042	-0.201, 0.145	0.754
	Psychological distress	0.143	-0.059, 0.177	0.336
	Everyday functioning	-0.103	-0.113, 0.027	0.234
	Weekly game time	0.139	-0.008, 0.065	0.129
	Onset	0.278	0.010, 0.384	0.062
	ADHD symptoms	**0.394**	**0.183, 0.560**	< 0.001*
	ASD symptoms	0.033	-0.079, 0.104	0.795
				Adjusted R-squared: 0.387
Model 2 Psychological distress	Age	0.383	0.319, 0.938	0.002*
	GD symptoms	0.042	-0.344, 0.546	0.658
	Everyday functioning	0.097	-0.044, 0.239	0.180
	Weekly game time	0.008	-0.065, 0.072	0.917
	Onset	**0.214**	**0.078, 0.676**	0.019*
	ADHD symptoms	**0.336**	**0.389, 1.123**	< 0.001*
	ASD symptoms	**0.176**	**0.032, 0.347**	0.023*
				Adjusted R-squared: 0.609
Model 3 Everyday functioning	Age	0.031	-0.343, 0.443	0.805
	Psychological distress	-0.129	-0.938, 0.315	0.333
	GD symptoms	0.221	-0.095, 0.526	0.180
	Weekly game time	**0.249**	**0.028, 0.217**	0.013*
	Onset	0.030	-0.446, 0.549	0.841
	ADHD symptoms	0.051	-0.476, 0.708	0.703
	ASD symptoms	0.196	-0.012, 0.419	0.070
				Adjusted R-squared: 0.149

GD, gaming disorder, measured with Gaming disorder test. Onset, onset of problematic gaming. Functioning, everyday functioning measured with GAF and CGAS. ADHD, attention deficit hyperactivity disorder, measured with ASRS. ASD, autism spectrum disorder, measured with RAADS-14.The * indicates that the p-value is statistically significant. The bold values are the values that are significant.

## 4 Discussion

The aim of this study was to examine the characteristics of 107 youth and adult patients seeking treatment for problematic gaming. By combining clinical interviews, standardized assessments, and self-report measures, we aimed to provide a more comprehensive understanding of this new group of patients. Overall, participants spent an average of 50 hours per week gaming, and 80% met the diagnostic criteria for gaming disorder, while the remainder exhibited hazardous gaming, reflecting a spectrum of gaming-related difficulties.

The sample reported low gaming disorder symptom burden, despite clinician-diagnosed gaming disorder in most cases. Among participants aged 13–15, only a minority exceeded self-report cut-off scores, whereas 95% of parents reported elevated symptoms. This suggests a discrepancy between participants’ self-perception and how their difficulties are perceived by others. In light of this discrepancy, it is also noteworthy that time spent gaming was not significantly associated with self-reported gaming disorder symptoms in the regression analyses. This finding supports previous research suggesting that gaming duration may be a poor standalone predictor of problematic gaming (28, 29). In contrast, the regression analyses identified self-reported ADHD symptoms as uniquely associated with gaming disorder symptoms, consistent with prior research suggesting that impulsivity and ADHD traits contribute to gaming-related problems (15, 30). Researchers have suggested that individuals with ADHD are more prone to gaming problems due to impulse-control difficulties and the design of video games (22, 30). A factor explaining this association is impulsivity, a core symptom of ADHD that can lead to difficulties in delaying gratification. Individuals with ADHD tend to prefer immediate over delayed rewards, making video games especially appealing. In addition, some propose that individuals with ADHD have a greater need for stimulation to achieve an optimal level of arousal, which may further increase the appeal of a stimulating activities such as gaming (15). These reinforcing qualities of games may not only encourage longer play sessions but also make it more difficult for individuals with ADHD to disengage from gaming even when it interferes with their daily life.

Psychological distress was relatively low in this group. Several non-clinical studies have found that gaming disorder is associated with high psychiatric comorbidity and psychological distress (31–34), but this pattern does not appear to hold in our clinical population. Only a third of participants reported moderate to severe psychological distress, and parent-child reports were consistent here. Clinician assessments also indicated low distress levels, with fewer than 20% meeting criteria for mood or anxiety disorders. Similar findings were reported by Granero, Fernández-Aranda (35) and partially by Torres-Rodríguez, Griffiths (18), in their sample of treatment-seeking patients. Regression analysis showed that psychological distress was associated with ADHD and ASD symptoms, older age, and later onset of problematic gaming. This aligns with previous research linking neurodevelopmental traits to poorer quality of life (36, 37). Younger participants might experience lower distress due to support from parents and schools, which can help maintain structured routines and buffer against distress. In contrast, those who develop problematic gaming later in life may lack such support systems, making them more vulnerable to distress and more affected by the consequences of their gaming behavior.

Substance use and other behavioral addictions, including problematic social media use and gambling, were relatively rare. Despite high screen time beyond gaming, participants reported low levels of problematic social media use. Gambling with money or in-game items was only slightly more common. Despite growing concerns about gambling elements in games, particularly loot boxes (38), only 7% had purchased loot boxes in the past month, and just one participant reported spending a large amount (650$) on in-game purchases. These findings support previous research suggesting that behavioral addictions are domain-specific, and that problematic gaming does not necessarily extend to other behavioral domains (39).

Participants in this sample demonstrated low everyday functioning, as rated by clinicians using the GAF and CGAS, reflecting a high degree of functional impairment. Many participants reported significant challenges in daily life, including limited offline social interactions and many adults living with their parents. In line with previous research (19, 40), among participants aged 20 and older, only half were employed or studying, while only a third of those aged 13–15 met their academic goals. Everyday functioning stands out in this sample, other variables examined were generally low and showed higher variability. In contrast, high functional impairment was high and had low variability, with 69% of participants exhibiting moderate to severe impairment, and 91% showed at least mild to moderate difficulties. This suggests that functional impairment likely is a defining characteristic of this group. Notably, the regression model examining everyday functioning explained little variance, with time spent gaming as the only significant variable. Neither psychological distress nor gaming disorder symptoms were significantly associated, suggesting a disconnect between self-reported symptoms and observable difficulties. This may reflect a limited insight and highlights the need for external assessments when evaluating this population.

To better understand why self-reported symptoms of gaming disorder differed from clinician assessments and how low symptom burden can coexist alongside high functional impairment, it is important to consider the psychological function of gaming in this population. Gaming may function both as an avoidance strategy to temporarily suppress negative emotions or avoid real-life problems (41, 42) as well as a means of fulfilling basic psychological needs (43). From the perspective of Self-Determination Theory (SDT), individuals may turn to games in order to fulfill their basic psychological needs for competence, relatedness, and autonomy, needs that games are particularly effective to meet (44). These needs can be fulfilled through game progression and skill development (competence), social interaction (relatedness), and the freedom to make meaningful choices within the game (autonomy). Because games provide easier access to feelings of competence, social connection, and autonomy compared to real-world settings, individuals may increasingly turn to gaming to meet these needs and avoid their problems. For individuals with lower everyday functioning, these needs may be harder to fulfill in other parts of life, making gaming an especially attractive alternative. Previous studies applying SDT to gaming have shown that individuals who experience basic psychological need deprivation in their everyday lives are more likely to engage in gaming in an obsessive and compulsive manner, using games to compensate for unmet psychological needs (45, 46). In contrast, those with high real-life need satisfaction tend to engage in gaming less problematic (45). The fact that many participants in our sample reported elevated symptoms of ADHD and ASD further supports this idea, as these neurodevelopmental conditions are often associated with difficulty meeting societal expectations (47). One study found that frustration of basic psychological needs can negatively affect self-control, which in turn contributes to problematic gaming. This may help explain the development of gaming disorder from a SDT perspective, especially among individuals with neurodevelopment disorders (48). Over time, this reliance may reduce engagement in everyday responsibilities and reinforce gaming as an avoidance-based coping mechanism. As gaming begins to displace other activities, individuals may lose opportunities to develop real-world skills, further cementing functional impairment rather than improving it. Meanwhile, subjective symptom levels may remain low, as individuals continue to avoid their problems and fulfill basic psychological needs through gaming. Thus, while gaming may reduce perceived distress in short term, it may also contribute to long-term dysfunction.

### 4.1 Implications

These findings have implications for how psychological treatments and clinical services are designed for individuals with gaming disorder. Given the central role of impaired everyday functioning, treatment should not only target gaming behavior but also help patients increase engagement in other areas of life. Involving the family system may provide additional support needed by both youth and adult patients. The low perceived symptom burden observed in this group suggests that traditional symptom-based assessments may underestimate the impact of problematic gaming and contribute to reduced motivation for change, underscoring the need to address insight and motivation in treatment. Finally, the average five-year duration before seeking help points to the importance of easy access to care.

### 4.2 Limitations

This study has several limitations. First, the sample size was relatively small and exclusively recruited in southern Sweden, which may limit the generalizability of the results. Additionally, the sample was predominantly male, reflecting the typical demographic of individuals with gaming disorder, but notably missing the experiences of females and other gender identities. Second, the use of self-report questionnaires may introduce a threat to internal validity, as self-reports could lead to underreporting or overreporting in this specific group (8). This may be reflected in the observed discrepancies between some subjective ratings and clinician assessments. However, this limitation was partly addressed by using both self-reports and clinician-administered assessments. Third, differences in the questionnaires administered across age groups could influence the comparability of results. While the wide age range provides a valuable perspective on gaming disorder across different life stages, it complicates the description of this as a cohesive group. Lastly, this study has an exploratory approach, which may increase the risk of false-positive results. Despite these limitations, the study offers important insights into the clinical characteristics of patients seeking treatment for gaming disorder.

### 4.3 Future research

To better understand gaming disorder, future research must move beyond survey-based studies conducted in predominantly healthy populations. Instead, there is a need for clinically focused studies that use clinician-rated and objective measures to complement self-report data. This is particularly important given the discrepancies observed here between self-rated symptom burden and clinician-assessed impairment. Further research should examine why individuals with a clinical diagnosis of gaming disorder can report low levels of subjective distress and symptom severity. Understanding this may help us understand the psychological mechanisms that sustain gaming behavior, such as limited insight, denial, or the protective function of gaming. One place to start with is to investigate the relationship between everyday functioning and problematic gaming, both in terms of how functional difficulties may contribute to the onset of gaming problems, and how gaming may in turn reinforce functional impairment. Finally, future studies should explore the role of social and family factors. Social difficulties and unmet needs for relatedness may contribute to excessive gaming. Investigating how family dynamics, peer relationships, and social competence influence gaming patterns could inform better treatments.

### 4.4 Conclusion

Despite growing research on gaming disorder, few studies have examined the clinical characteristics of patients seeking treatment. This study contributes to that gap by providing a comprehensive description of this population. Self-reported symptoms of gaming disorder and psychological distress were relatively low, while functional impairment was substantial and emerged as a defining clinical characteristic of this group. We propose that gaming may be associated with both a low symptom burden and impaired everyday functioning. Gaming can reduce subjective distress by serving as an avoidance strategy and fulfilling basic psychological needs. However, it may displace other meaningful activities, thereby maintaining or even worsening functional impairment. These findings highlight that relying solely on symptom-based assessments may underestimate the impact of problematic gaming. Given the central role of impaired functioning, it should be addressed in psychological treatments. Patients need support not only in managing their gaming behavior but also in improving their functioning in key areas of life, such as school, work, and relationships.

## Data Availability

The raw data supporting the conclusions of this article will be made available by the authors, without undue reservation.
