# Supplementary material for: Who seeks treatment for gaming? Characteristics of young and adult patients seeking treatment for gaming disorder
Source: Front Psychiatry. 2025 Aug 12;16:1629932. doi: 10.3389/fpsyt.2025.1629932 (PMC12378942; doi:10.3389/fpsyt.2025.1629932)
Supplement: Supplementary file 1 [file DataSheet1.docx]

**Supplemental table and figures**

CONSORT diagram


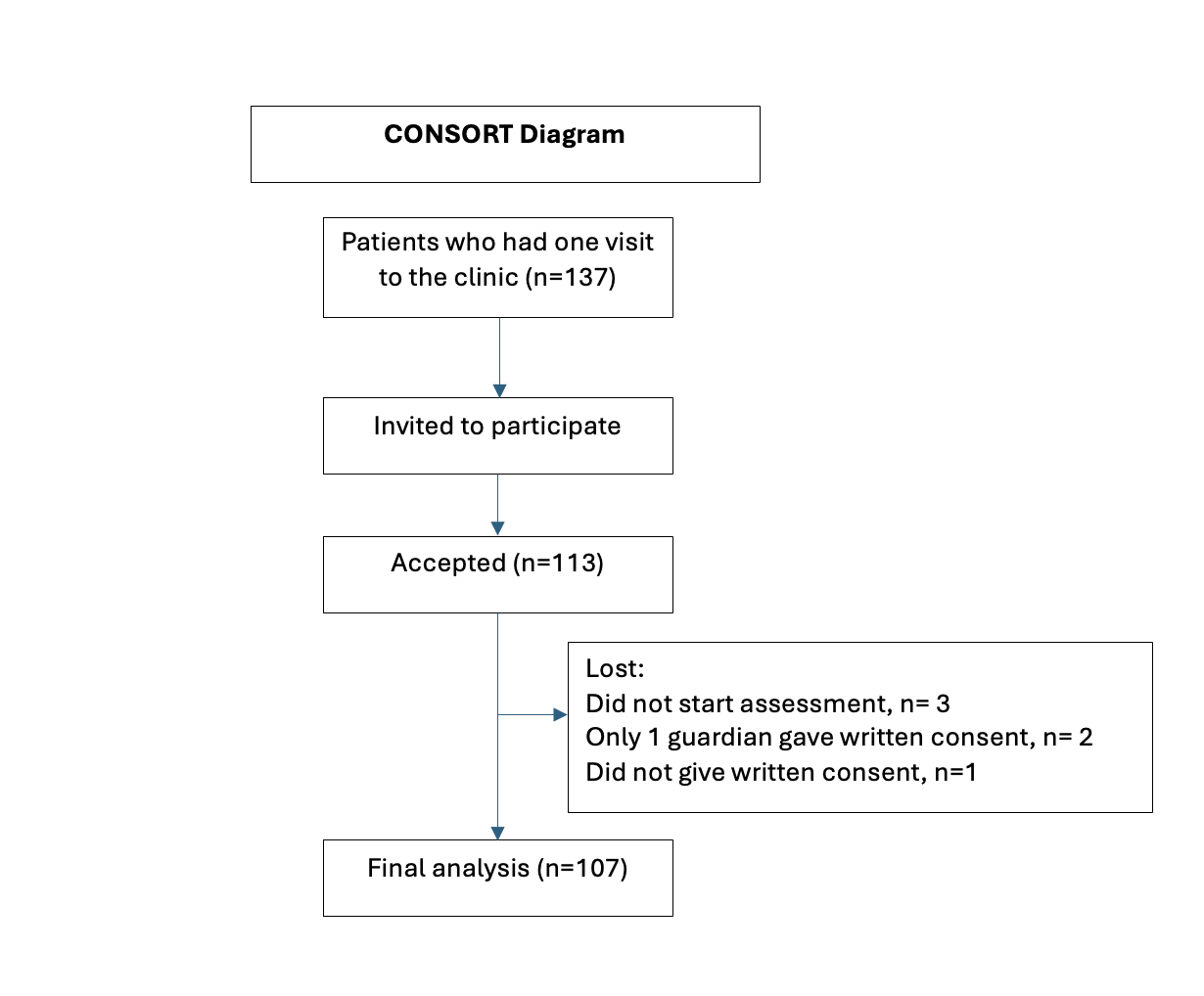


Supplemental table

*Games played by participants stratified by genre and monetization strategy*

| Model / Game Genre | Competitive | Story-driven | Simstrat | MMOE | Casual |
| --- | --- | --- | --- | --- | --- |
| Free-to-Play | Counterstrike  League of Legends  Valorant  Fortnite  Apex Legends  Overwatch  Rocket League  Rainbow 6  Fall Guys  Warframe |  | People’s Underground | World of Warcraft  Minecraft  Clash of Clans  Lost Ark  Runescape  Destiny  Path of Exile | Chess  Roblox  Pokemon Go  Tetris  Diggy’s A |
| Pay-to-Play | Call of Duty  PUBG  Dota  Risk  Serious Sam  Escape from Tarkov | Elden Ring  Skyrim  Baldur’s Gate  Witcher  Fallout  Hitman | Civilization  Rimworld  City Building  Crusader King  Terraria  Farming Simulator Monster Hunter  Entropy  DRG  Don’t Starve | No Man’s Sky  Ark Survival  Diablo  Lethal Company  7 Days to Die  Rust  Five M  Fountain of Youth  Star Wars Outlaws  Tibia  Sea of Thieves  Day Z | Five Nights at Freddy’s  Gary’s Mod |
